# FOXM1 inhibition primes terminal differentiation of human iPSC-derived hepatocytes

**DOI:** 10.1038/s41420-026-03178-9

**Published:** 2026-07-08

**Authors:** Kayque Alves Telles-Silva, Lara Pacheco, Sabrina Komatsu, Fernanda Chianca, Gustavo Chagas, Gabrielly Cristine Martins, Maria Gridina, Daria Panchenko, Valdemir Melechco Carvalho, Elia G. Caldini, Veniamin S. Fishman, Michelle Arkin, Ernesto Goulart, Mayana Zatz

**Affiliations:** 1 Human Genome Research Center (HUG-CEL), Department of Genetics and Evolutionary Biology, Institute of Biosciences, University of São Paulo, São Paulo, SP Brazil; 2 Department of Pharmaceutical Chemistry, University of California, San Francisco, San Francisco, CA USA; 3 Department of Hematology and Oncology, Federal University of São Paulo, São Paulo, SP Brazil; 4 The Federal Research Center Institute of Cytology and Genetics, Novosibirsk, Russia; 5 Sirius University for Science and Technology, Sochi, Russia; 6 Division of Research and Development, Fleury Group, São Paulo, Brazil; 7 Laboratory of Cellular Biology, Department of Pathology, Medical School, University of São Paulo, São Paulo, SP Brazil

**Keywords:** Stem-cell differentiation, Apoptosis

## Abstract

Human iPSC-derived hepatocytes are widely used in disease modeling. However, late embryonic development in the human liver remains elusive, which hinders differentiation. During late liver embryonic development, Topoisomerase II (TOP2) is downregulated; however, its role in differentiation is unclear. We replicated the TOP2 silencing at birth and identified a transcription factor crucial for hepatocyte differentiation in vitro. Subtoxic inhibition of TOP2 reduces nuclear chromatin condensation without causing DNA damage. RNA-seq analysis revealed that TOP2 inhibition induced cell cycle arrest, accompanied by FOXM1 downregulation. ATAC-seq confirmed that TOP2A inhibition decreased chromatin accessibility and modulated the Wnt/β-catenin pathway. Proteomic analysis demonstrated that FOXM1 inhibition mimicked TOP2A-mediated cell cycle arrest and reduced the levels of fetal hepatocyte proteins. Prolonged FOXM1 inhibition results in increased hepatocyte polyploidization, enhanced CYP450 activity, and improved lipid metabolism. Our findings suggest that FOXM1 inhibition promotes terminal differentiation of human iPSC-derived hepatocytes, potentially paving the way for understanding late embryonic development in the human liver.

**Figure:**
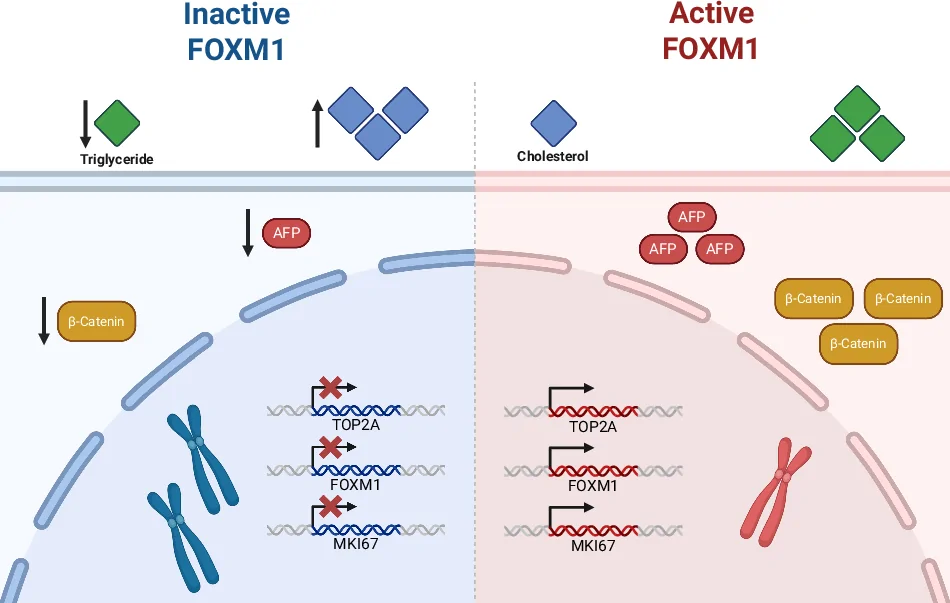

## Introduction

Human induced pluripotent stem cells (iPSCs) have emerged as a tool to bridge developmental biology, regenerative medicine, and disease modeling because of their capacity to differentiate into various cell types, including hepatocytes [1]. The need for a viable hepatocyte source for drug testing has driven the development of new techniques for hepatic differentiation [2]. Hepatocyte differentiation from iPSCs requires specific factors (Wnt3a, Activin A, HGF) and signaling pathways that control cell proliferation and maturation [3]. iPSC-derived hepatocytes show a fetal-like phenotype; thus, they are frequently named hepatocyte-like cells (HLCs), with a proliferative profile and lack of mature metabolism compared to primary human hepatocytes (PHHs) [4]. Therefore, no protocol has achieved terminal differentiation of iPSCs into hepatocytes using tridimensional, coculture-based, organoid, or microfluidic platforms [5].

Topoisomerases resolve DNA structures and TOP2 is crucial for DNA replication during embryogenesis [6]. TOP2 regulates hepatocyte proliferation [7]. Among the TOP2A and TOP2B isoforms, TOP2A upregulation facilitates oncogenesis by enhancing cellular proliferation [8]. TOP2A downregulation after weaning coincides with hepatocyte terminal maturation when the liver metabolism changes [9], revealing TOP2A’s role in liver development [10]. TOP2A and FOXM1 are frequently associated with hepatocyte proliferation studies [11, 12]. FOXM1 regulates cell cycle progression and TOP2A expression during embryogenesis and cancer development [13]. The TOP2-FOXM1 interaction maintains a balance between stemness and lineage commitment [14]. During late embryonic development, liver parenchymal cells stop proliferating and significantly increase their functionality, especially hepatocyte polyploidization, which is crucial for liver homeostasis [15]. Foxm1-deficient mice exhibit reduced hepatocyte mitosis and polyploidy [16], suggesting that FOXM1 may be involved in the regulation of hepatocyte cell cycle and polyploidization. However, the TOP2-FOXM1 axis has not been explored in the context of hepatocyte terminal differentiation.

In this study, we investigated the effects of TOP2 and FOXM1 on the differentiation of human iPSC-derived hepatocytes. FOXM1 inhibition promotes hepatocyte maturation, including cell cycle arrest and polyploidy. FOXM1 suppression reduces fetal markers while increasing adult hepatic gene expression by modulating the Wnt/β-catenin pathway. TOP2A functions as an integral partner of this network.

## Results

### Subtoxic etoposide treatment promotes quiescence of HLCs

**Fig. 1:**
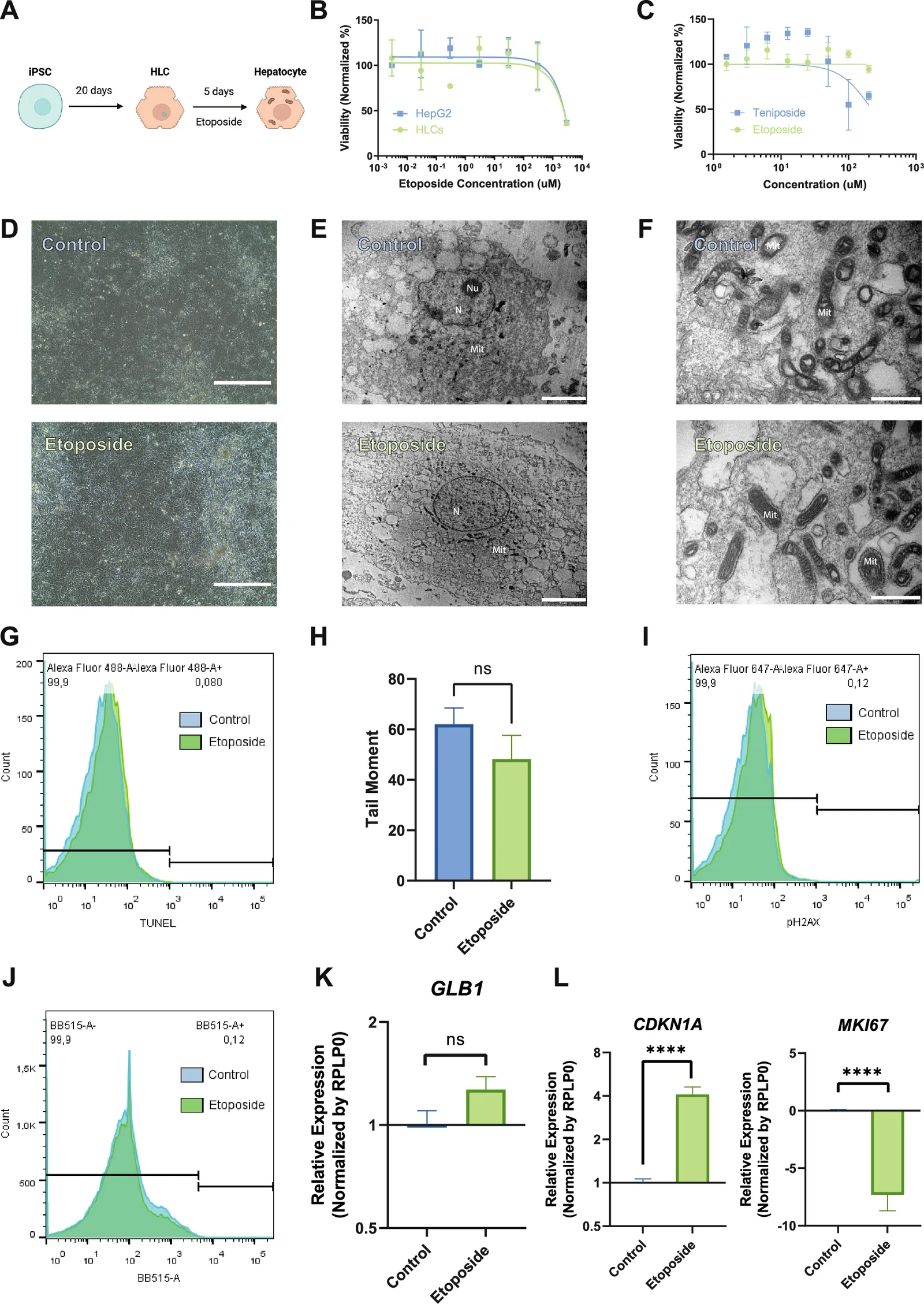
Low-dose etoposide preserves the viability and DNA integrity of HLCs. **A** Schematic of etoposide treatment of HLCs (Created using BioRender.com). **B** Cell viability assay of HLCs and HepG2s negative control) with different concentrations of etoposide to determine toxic doses (*n* = 2 biological replicates and 3 independent experiments for HLCs, *n* = 3 independent experiments for HepG2). **C** Cell viability assay of HLCs with different concentrations of etoposide and teniposide to compare toxic doses. **D** Brightfield images of HLCs treated with 10 μM etoposide (scale bar = 400 μm). **E** Transmission electron micrograph of etoposide-treated HLCs (scale bar = 5 μm). N = nucleus; Nu = nucleoli; Mit = mitochondria. **F** Transmission electron micrograph of etoposide-treated HLCs’s mitochondria (scale bar = 1 μm). Mit = mitochondria. **G** TUNEL assay of etoposide-treated HLCs. **H** COMET assay of etoposide-treated HLCs. **I** Flow cytometry of pH2AX staining of etoposide-treated HLCs. **J** Flow cytometry of hydrolyzed β-galactosidase staining of etoposide-treated HLCs. **K** RT-qPCR of *GLB1* of etoposide-treated HLCs. **L** RT-qPCR of *CDKN1A* and *MKI67* of etoposide-treated HLCs. In every experiment, *n* = 2 biological replicates (7007 and 7405 cell lines) and 3 independent experiments. In **B**, **C**, **H**, **K**, and **L**, data is mean ± SD. In **B** and **C**, paired t test (p < 0.05). In **H**, **K**, and **L**, t test (p < 0.001).

To assess the effect of etoposide on TOP2 inhibition in HLCs, human iPSCs were differentiated into HLCs (Supplementary Fig. S1A). The HLCs exhibited hepatocyte morphology on day 20 (Supplementary Fig. S1B) and more than 71.4% double-positive staining for ALB and UGT1A1 (Supplementary Fig. S1C) and significant hepatocyte-specific gene expression (*HNF4A*, *ALB*, and *CYP3A4*) (Supplementary Fig. S1D). To determine its subtoxic concentrations, we exposed HLCs to etoposide for five days (Fig. 1A), according to administration standards [16, 17]. Etoposide did not induce apoptosis at concentrations of up to 3 mM, with similar responses observed in HLCs and HepG2 cells. (Fig. 1B). We also validated apoptosis using teniposide, which is nontoxic at low concentrations (Fig. 1C). Therefore, 10 µM of etoposide was selected for further experiments. Etoposide-treated cells showed polygonal morphology similar to that of primary human hepatocytes (PHHs) (Fig. 1D). Similar to adult PHH organoids, etoposide-treated HLCs lacked strong nucleolar staining (Fig. 1E). At 10 µM, etoposide increased mitochondrial cristae elongation (Fig. 1F), resembling metabolically active PHHs, whereas control HLCs showed mitochondrial vacuolization. The TUNEL and comet assays revealed no significant differences in DNA damage between the treated and control groups (Fig. 1G and H). Flow cytometric analysis of H2AX phosphorylation showed no differences between groups (Fig. 1I). These results confirmed that 10 µM etoposide is non-toxic and does not cause DNA damage in HLCs.

We further evaluated the effect of 10 µM etoposide on HLC senescence by measuring the β-galactosidase activity using flow cytometry and RT-qPCR. No significant differences were observed between the treated and control groups (Fig. 1J, K), indicating that etoposide does not induce senescence. Quiescence is relevant in iPSC-derived hepatocytes, marking terminal differentiation in disease modeling and drug development [18]. *CDKN1A* expression increases during both cellular senescence and quiescence, whereas *MKI67* expression is down-regulated in quiescent hepatocytes. RT-qPCR showed that 10 µM etoposide increased *CDKN1A* expression and decreased *MKI67* expression (Fig. 1L). Overall, 10 µM etoposide did not induce apoptosis, DNA damage, or senescence in HLCs but potentially induced quiescence.

### RNA-Seq reveals etoposide-induced transcriptome rewiring and cell cycle arrest in HLCs

**Fig. 2:**
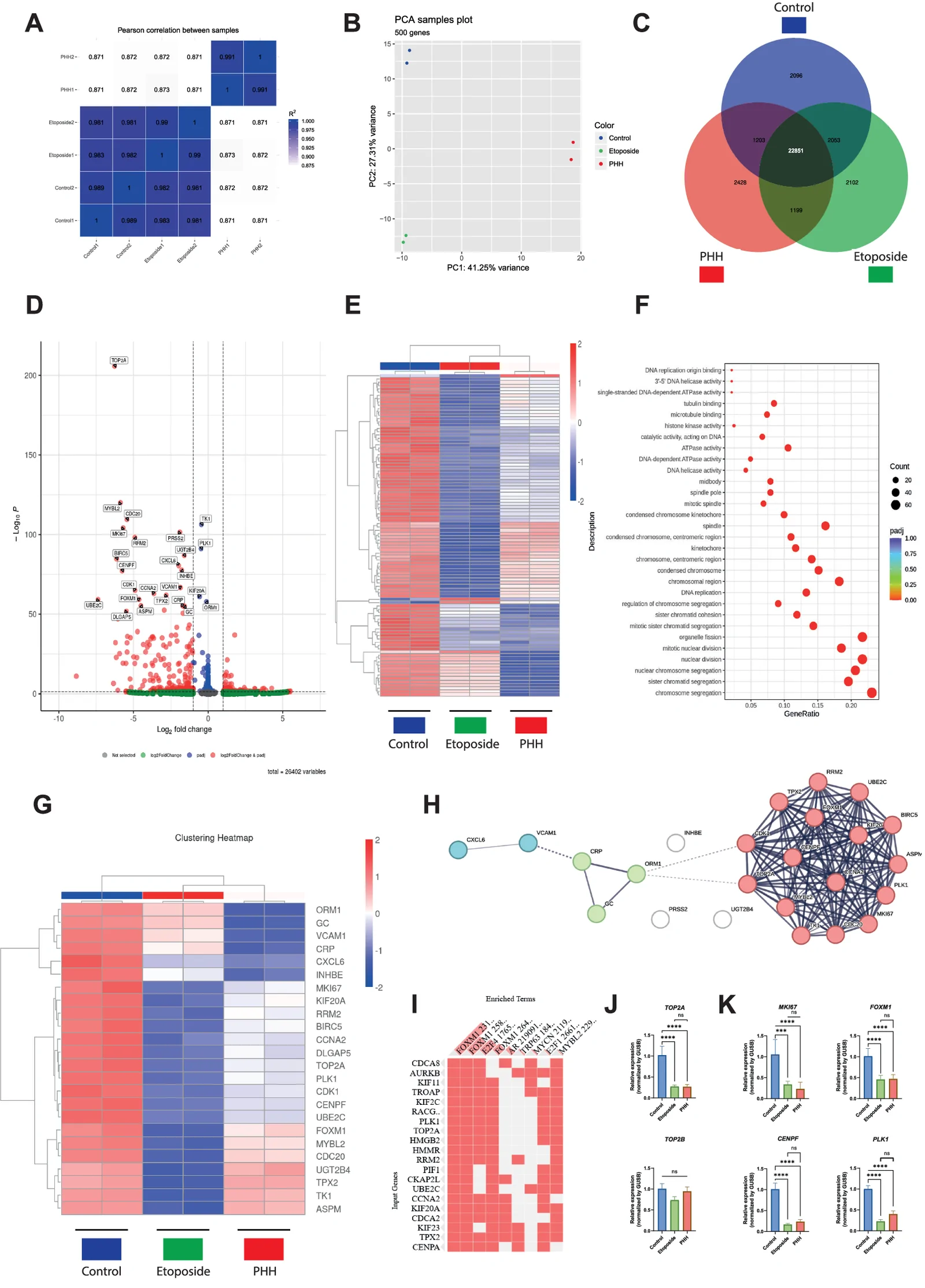
RNA-seq analysis of etoposide-treated HLCs. **A** Inter-sample correlation matrix heatmap (R^2^ = square of Pearson correlation coefficient) of control HLCs, etoposide-treated HLCs, and primary human hepatocytes (PHHs). **B** Principal component analysis (PCA) on fragments per kilobase per million mapped fragments (FKPM). **C** Venn diagram on all identified genes. **D** Volcano plot of differentially expressed genes between etoposide-treated and control HLCs (|log2(FoldChange)| >= 1 & padj <= 0.05). **E** Heatmap of the top 100 z-scored differentially expressed genes between etoposide-treated and control HLCs. **F** Gene ontology (GO) scatter plot for biological processes of the top 100 z-scored differentially expressed genes between etoposide-treated and control HLCs. The size of the circle indicates the percentage of genes in each annotation category. **G** Heatmap of the top 25 z-scored differentially expressed genes between etoposide-treated and control HLCs. **H** STRING interactome of the top 25 differentially expressed genes between etoposide-treated and control HLCs. The colors represent k-means clustering. **I** ChIP-X enrichment analysis (ChEA) of the top 25 differentially expressed genes between etoposide-treated and control HLCs. RT-qPCR of *TOP2A* and *TOP2B* (**J**); or *MKI67*, *FOXM1*, *CENPF*, and *PLK1* (**K**) of etoposide-treated HLCs. In every experiment, *n* = 2 biological replicates (WTC-11 and 7007 cell lines). In every experiment, etoposide was used at 10 μM for five days. In **E** and **G**, the color represents the average expression level for the indicated gene. In J and K, data is mean ± SD, analyzed by one-way ANOVA with multiple comparisons and Tukey’s correction (p < 0.001); *n* = 2 biological replicates (WTC-11 and 7007 cell lines) and 3 independent experiments.

Low-dose etoposide facilitates osteogenesis in certain cell lines [19] and shifts apoptotic pathways from caspase-dependent to differentiation-linked [20]. To further evaluate the effects of etoposide on HLCs, RNA sequencing was performed. Intra-group correlations were higher than inter-group correlations, with control and treated HLCs being similar to PHHs (Fig. 2A). PCA showed that etoposide changed the expression profile of HLCs compared to that of the controls (Fig. 2B), with PC1 accounting for cell source variance (41.25%) and PC2 accounting for etoposide treatment (27.3%). A Venn diagram revealed that 3,301 genes were exclusively expressed in etoposide-treated HLCs (Fig. 2C). Etoposide induced the downregulation of global gene expression (Fig. 2D), with *TOP2A* being the most downregulated. Although etoposide inhibited both TOP2A and TOP2B isoforms, *TOP2B* expression remained unchanged at 10 µM. Among the 1,010 differentially expressed genes, etoposide-induced changes made HLCs resemble PHHs (Fig. 2E). These genes are associated with chromosome segregation, nuclear division, and cell cycle arrest (Fig. 2F). Although etoposide typically causes cell cycle arrest via DNA damage [21], this was not observed in HLCs. Analysis showed that etoposide-treated HLCs resembled PHHs more than control HLCs (Fig. 2G), with the downregulation of cell cycle progression genes. Seventeen genes formed the predicted protein interaction core (Fig. 2H). ChEA analysis identified FOXM1 and E2F4 as the main enriched transcription factors (Fig. 2I). FOXM1 regulates cell cycle progression [22], suggesting an involvement of FOXM1 in the maturation of HLCs.

Next, to characterize the cell cycle arrest caused by etoposide in HLCs, we found that 10 µM etoposide showed isoform specificity and reduced *TOP2A*, but not *TOP2B*, gene expression (Fig. 2J). Cell cycle arrest was confirmed by reduced *MKI67* and *FOXM1* expression levels, which were similar to those observed in PHHs. Downregulation of *CENPF* and *PLK1* expression (Fig. 2K) was correlated with hepatocyte polyploidization during embryonic liver development [23], indicating that TOP2A inhibition may induce cytokinesis failure and hepatocyte polyploidization. Overall, RNA-seq indicated that treating HLCs with etoposide shifted their transcriptional profile towards PHHs, especially regarding cell cycle regulation gene sets.

### TOP2A inhibition remodels chromatin architecture to suppress Wnt/JAK-STAT signaling and cell cycle gene expression

**Fig. 3:**
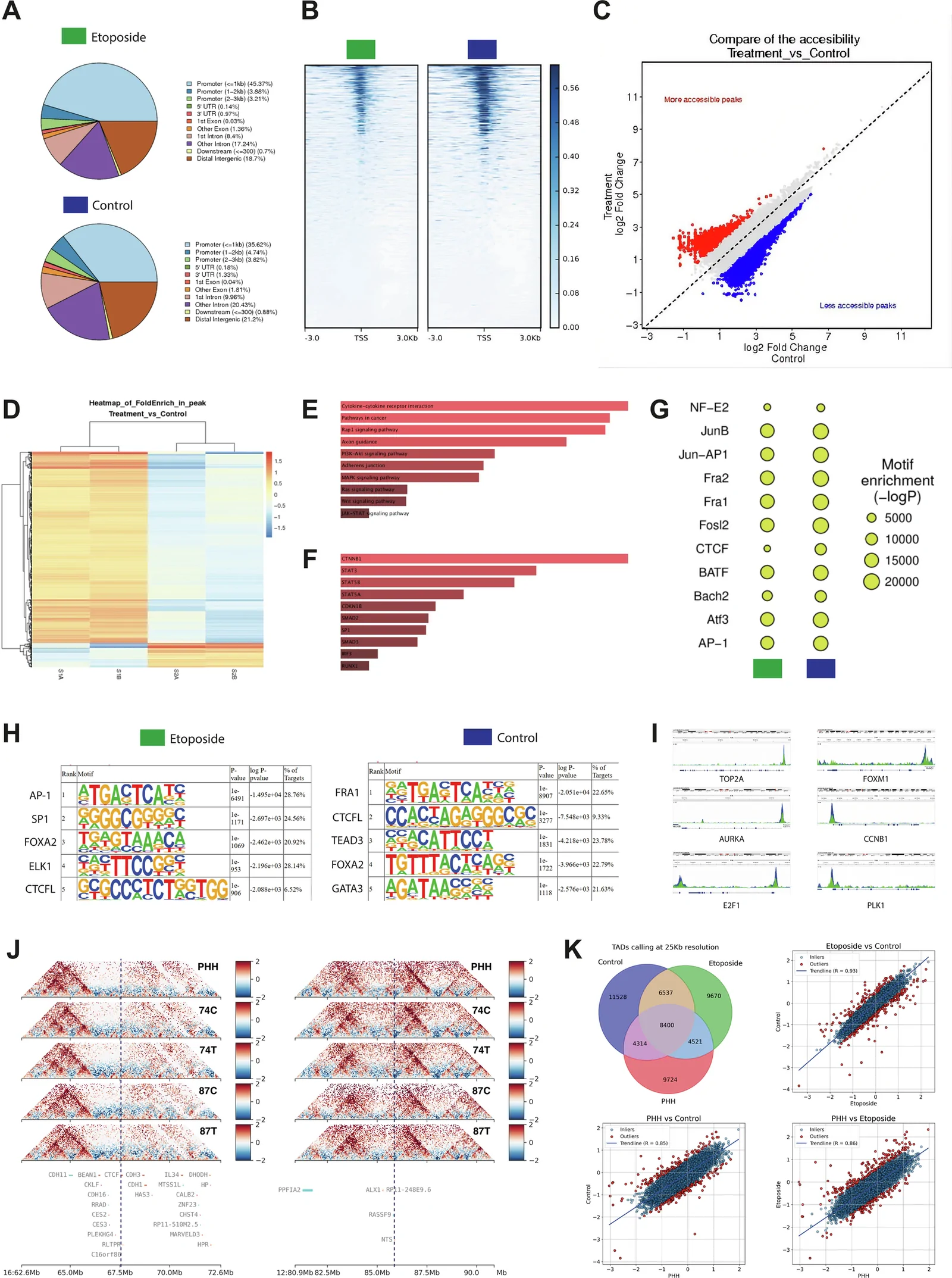
ATAC-seq and Hi-C analysis of etoposide-treated HLCs. **A** Peak distribution in functional gene regions of control and etoposide-treated HLCs. **B** Distribution of the reads mapped to the TSS. The color indicates the average density of reads. **C** Comparison of global chromatin accessibility. Treatment, etoposide-treated HLCs; Control, control HLCs. **D** Heatmap of z-scored peak fold enrichment between etoposide-treated and control HLCs. The color represents the average normalized enrichment per chromatin domain. **E** KEGG analysis of the top 100 enriched peak chromatin domains between etoposide-treated and control HLCs. **F** Transcription factor protein-protein interaction (PPI) analysis of the top 100 enriched peak chromatin domains between etoposide-treated and control HLCs. **G** Enrichment of motif and known transcription factor motif. The size of the circle indicates the average degree of enrichment of the motif’s motif and the corresponding transcription factor’s motif. **H** Motif analysis of the conserved sequence of the peak enrichment position. The motifs are averaged by control or etoposide-treated HLCs and ranked by *p* value. **I** Chromatin accessibility analysis of *TOP2A*, *FOXM1*, *AURKA*, *CCNB1*, *E2F1*, and *PLK1* genes. The peaks represent the average pileup signal for control (blue) or etoposide-treated (green) HLCs. **J** Chromatin architecture analysis. Hi-C contact maps (observed/expected) at 50 kb resolution for primary human hepatocytes (PHH), control (74 C, 87 C), and etoposide-treated (74 T, 87 T) HLCs. Left, *CTCF* genomic region; Right, *NTS* genomic region. **K** Topologically associating domains (TADs) and insulation scores. Upper left, venn diagram of shared and condition-specific TAD boundaries identified at 25 kb resolution. Upper right and bottom, pairwise comparisons of insulation scores at TAD boundaries between samples. Each point represents an individual TAD boundary; Red dots, outliers with inslation score differences greater than three standard deviations; Blue lines, linear regression trend lines and Pearson correlation coefficient. In every experiment, *n* = 2 biological replicates (7405 and 8799 cell lines). In every experiment, etoposide was used at 10 μM for five days. Blue, control HLCs; Green, etoposide-treated HLCs. In **E** and **F**, the color and length of the bars indicate the combined ranked *p* value and odds ratio.

To assess the effect of TOP2A inhibition on chromatin accessibility, we conducted ATAC-seq on etoposide-treated HLCs and their controls. Etoposide induces global chromatin remodeling and transcriptional silencing [24]. In etoposide-treated HLCs, we observed an increase in short-range promoter peak identification (Fig. 3A) but a decrease in overall chromatin accessibility (Fig. 3B). The volcano plot illustrates this reduced chromatin accessibility (Fig. 3C), which accounts for the transcriptional repression observed in the RNA-seq analysis (Fig. 2D). Hierarchical clustering revealed a clear distinction between the treated and control groups, with treated HLCs exhibiting less accessible peaks (Fig. 3D) aligned with gene silencing (Fig. 2E). KEGG analysis indicated alterations in Wnt and JAK-STAT pathways (Fig. 3E), which are crucial for fetal hepatocyte proliferation [10]. The PI3K-Akt pathway influences the effects of Wnt signaling [25]. After treatment, chromatin accessibility to PI3K-Akt pathway effectors increased (Fig. 3F). CTCF, which is linked to Wnt signaling [26], emerged as the most differentially enriched motif between the groups (Fig. 3G). Changes in CTCFL expression suggested reduced binding (Fig. 3H). IGV visualization confirmed the decreased accessibility of gene promoters related to hepatocyte proliferation and polyploidization (Fig. 3I).

TOP2B interacts with CTCF and influences gene expression through its topological structure [27]. In HLCs, 10 μM etoposide inhibited TOP2A, but not TOP2B, and Hi-C analysis of differentially expressed genes revealed no significant changes in chromatin contacts (Fig. 3J), suggesting that expression changes were independent of genome architecture. TAD and loop analyses demonstrated high similarity across samples, with HLCs and controls exhibiting greater boundary and loop strength concordance (6537) than PHHs (4314 in control, 4521 in treated) (Fig. 3K). The PHHs displayed stronger loop anchor contacts (p = 0.016) (Supplementary Fig. S2B), and a more distinct compartmentalization beyond 40 Mb (Supplementary Fig. S2D). These findings indicate that etoposide reduced chromatin accessibility and facilitated transcriptional cell cycle arrest via Wnt signaling while preserving the PHH 3D landscape regardless of treatment.

### FOXM1 modulates β-catenin signaling and suppresses fetal markers

**Fig. 4:**
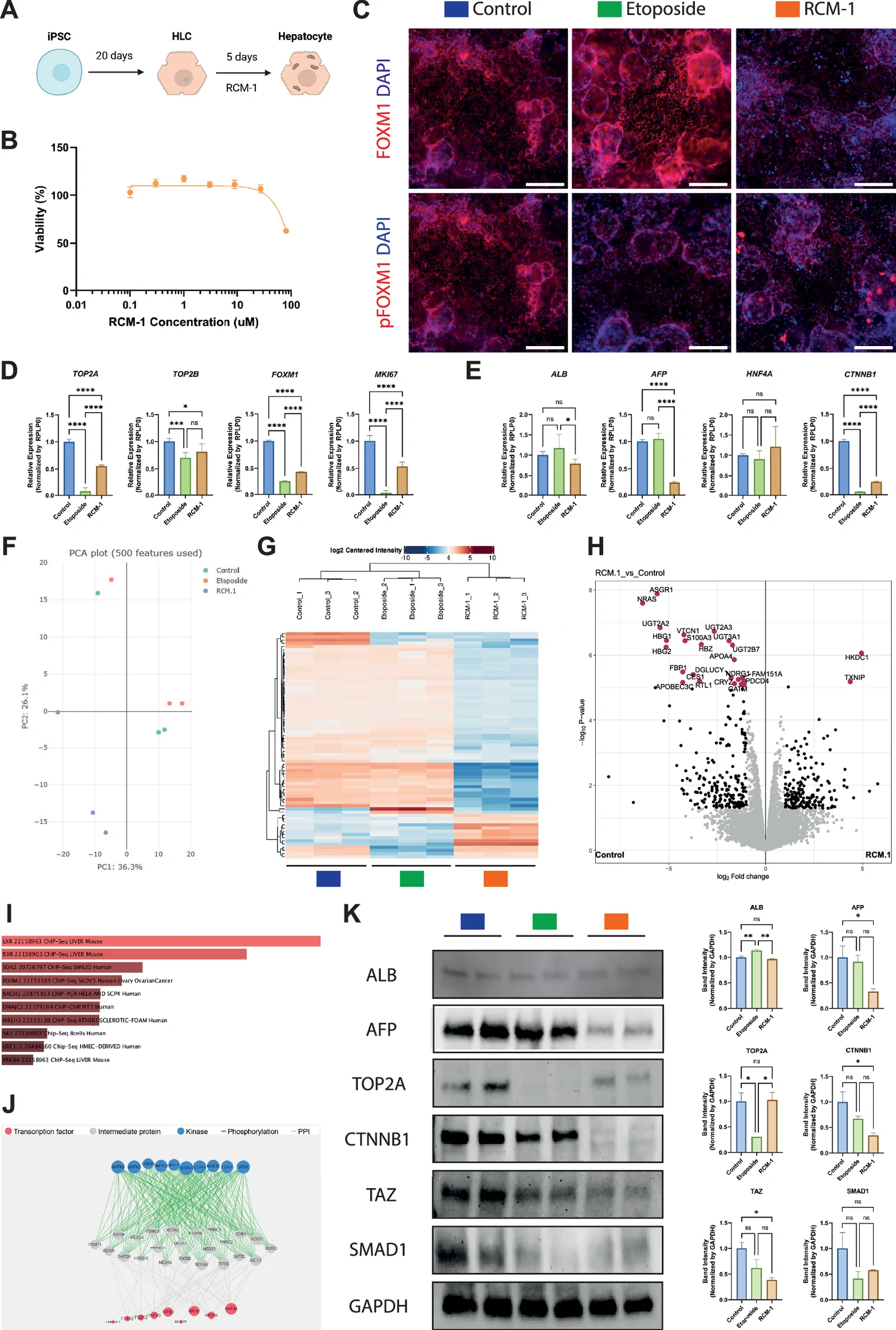
Phosphoproteomic analysis of RCM-1- and etoposide-treated HLCs. **A** Schematic of RCM-1 treatment of HLCs (Created using BioRender.com). **B** Cell viability assay of HLCs with different concentrations of RCM-1 to determine toxic doses. **C** Immunostaining for FOXM1 or phosphorylated Thr600 FOXM1 (pFOXM1) (Red) and DAPI (Blue) (scale bar = 300 μm). RT-qPCR of *TOP2A*, *TOP2B*, *FOXM1*, and *MKI67* (**D**); or *ALB*, *AFP*, *HNF4A*, and *CTNNB1* (**E**) of control, etoposide- and RCM-1-treated HLCs. **F** Principal component analysis (PCA) on total identified peptides (21,353), adjusted for 500 features. **G** Heatmap of the top 100 z-scored differentially abundant proteins between all samples. The color represents the average abundance level of each protein. **H** Volcano plot of differentially abundant proteins between RCM-1-treated and control HLCs (|log2(FoldChange)| >= 1 & FDR adjusted *p* value <= 0.05). **I** ChEA analysis of the top 100 differentially abundant proteins between RCM-1-treated and control HLCs. The color and length of the bars indicate the combined ranked *p* value and odds ratio. **J** Expression2Kinases (X2K) analysis of the top 100 differentially abundant proteins between RCM-1-treated and control HLCs. The circle size indicates the number of differentially abundant targets of each transcription factor or kinase. **K** Representative western blotting gels for ALB, AFP, TOP2A, CTNNB1, YAP/TAZ, SMAD1, and GAPDH. Blue, control HLCs; Green, etoposide-treated HLCs; Orange, RCM-1-treated HLCs. In **B**–**E**, and **K**, *n* = 2 biological replicates (7007 and 7405 cell lines) and 3 independent experiments. In **F**–**H**, *n* = 3 biological replicates (7007, 7405, and 8799 cell lines). In every experiment, etoposide was used at 10 μM and RCM-1 was used at 1 μM for five days. In **B**, **D**, **E**, and **K**, data is mean ± SD. In **D**, **E**, and **K**, one-way ANOVA with multiple comparisons and Tukey’s correction (p < 0.05).

FOXM1 regulates TOP2A expression and interacts with beta-catenin [28]. We administered RCM-1, an extensively characterized potent and specific inhibitor of FOXM1, to HLCs for five days (Fig. 4A), according to administration standards and paired with the experimental design used for etoposide. RCM-1 is known to reduce cell proliferation and induce apoptosis [29]. The CellTiter-Glo assay indicated that concentrations exceeding 30 µM diminished the HLC viability (Fig. 4B). At a concentration of 1 µM RCM-1 (chosen for all subsequent assays), immunofluorescence staining demonstrated a reduction in both FOXM1 and phosphorylated Thr600 FOXM1 (pFOXM1) levels (Fig. 4C). The HLCs treated with etoposide exhibited decreased pFOXM1 levels, suggesting impaired nuclear translocation. In HLCs treated with RCM-1, *TOP2A*, but not *TOP2B* was significantly reduced, even though *FOXM1* and *MKI67* were reduced in both etoposide- and RCM-1-treated HLCs (Fig. 4D). Following treatment with etoposide and RCM-1, the expression of *FOXM1* and *MKI67* decreased. While *ALB* and *HNF4A* levels remained stable, *AFP* expression decreased with FOXM1 inhibition. Both treatments decreased *CTNNB1* expression (Fig. 4E), indicating cell cycle arrest through FOXM1 modulation.

To further investigate the effects of FOXM1 inhibition, phosphoproteomic analysis revealed that etoposide and RCM-1 formed distinct clusters compared with the controls (Fig. 4F). RCM-1 induced a more significant downregulation of proteins than etoposide and controls (Fig. 4G). We identified 90 differentially abundant proteins, most of which were down-regulated (Fig. 4H). The top proteins include fetal hemoglobins (HBG1 and HBG2), UGT2B7, and ASGR1, which are expressed in fetal tissues [30–32]. ChEA analysis highlighted enriched LXR and RXR terms (Fig. 4I) that are crucial for adult hepatocyte lipid metabolism, detoxification, and differentiation [33]. The X2K aptamer identified HNF4A/G and FOXA2 as transcription factors, RXRA/B/G and TP53 as intermediate proteins, and GSK3B and ATM as kinases (Fig. 4J). These factors are involved in regulating liver development, proliferation, and differentiation [25, 34, 35], suggesting a role for FOXM1 in the liver development pathways.

Next, we analyzed protein abundance using western blotting to validate qPCR and phosphoproteomic findings. FOXM1 inhibition maintained ALB protein levels while reducing AFP levels (Fig. 4K), consistent with gene expression patterns (Fig. 4E). Although RCM-1 reduced TOP2A expression (Fig. 4D), the TOP2A protein levels remained unchanged. β-catenin abundance decreased after TOP2 inhibition, and was depleted by FOXM1 inhibition (Fig. 4K). Wnt signaling enhances YAP/TAZ activity in fetal hepatocytes, whereas the Hippo pathway suppresses YAP/TAZ activity in adult hepatocytes to maintain differentiation. Thus, we evaluated TAZ and SMAD1 protein abundance and identified a significant reduction in TAZ upon RCM-1 treatment (Fig. 4K). Overall, FOXM1 modulated the β-catenin pathway and suppressed hepatocyte fetal markers, potentially promoting hepatocyte maturation.

### FOXM1 inhibition promotes metabolic maturation and polyploidization of HLCs

To assess the long-term effects of TOP2A and FOXM1 inhibition on hepatocyte maturation, HLCs were treated with etoposide and RCM-1 until day 41 of differentiation (D41) (Supplementary Fig. S3A). The lactate dehydrogenase (LDH) release assay revealed no significant cytotoxicity (Supplementary Fig. S3B). In D41 HLCs, *TOP2A*, *FOXM1*, and *MKI67* were significantly downregulated (Supplementary Fig. S3C), indicating cell cycle arrest. *ALB* expression increased with TOP2A inhibition, whereas *AFP* expression decreased under both conditions (Supplementary Fig. S3D). *HNF4A* was also significantly downregulated, suggesting the loss of fetal characteristics and induction of adult hepatic function, as the HNF4A P2 isoform decreases postnatally [36]. *E2F1* and *E2F8* polyploidization markers that were decreased in adult hepatocytes [36] were downregulated by FOXM1 inhibition but not by TOP2A (Supplementary Fig. S3E).

**Fig. 5:**
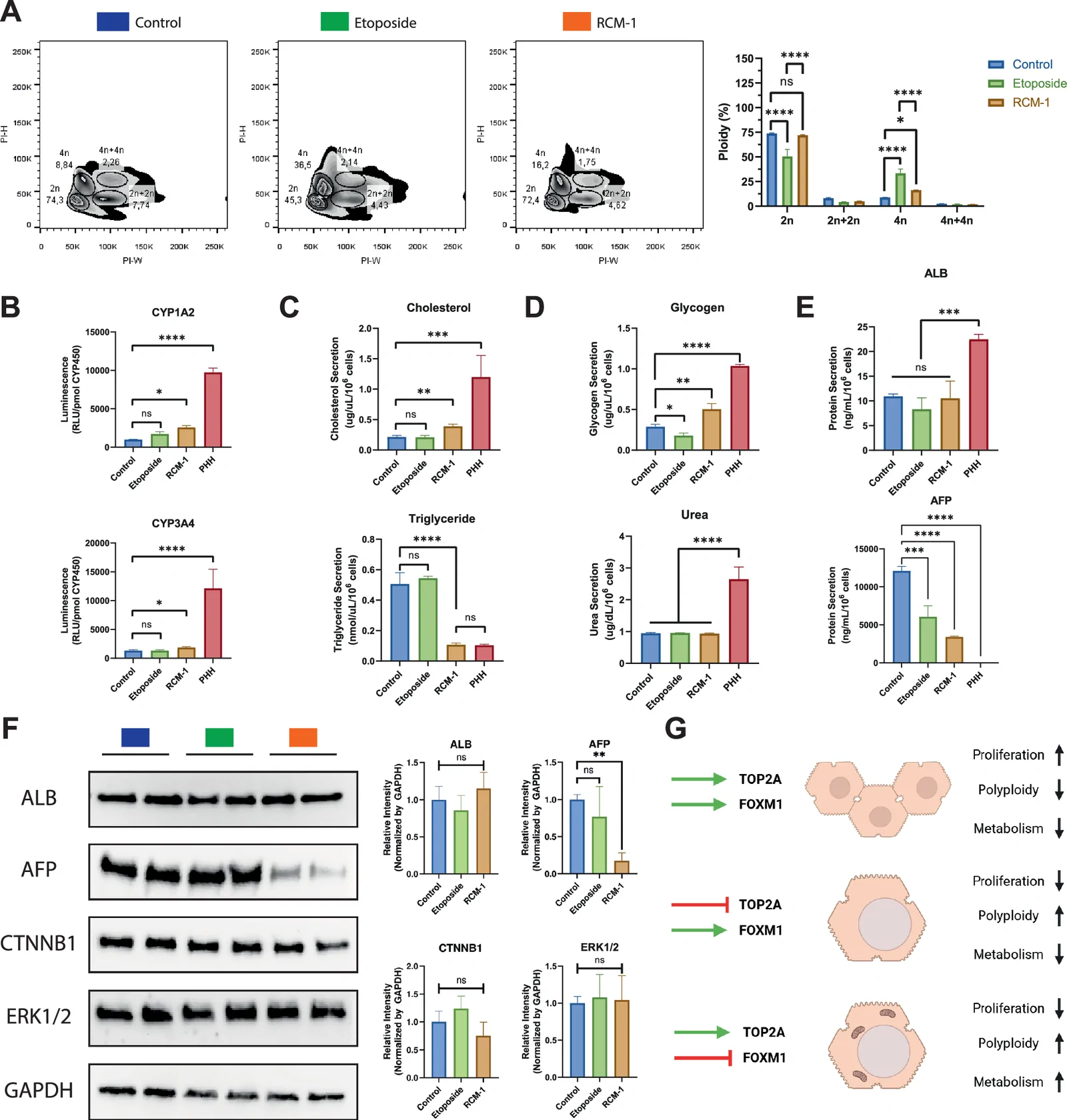
FOXM1 inhibition promotes metabolic maturation and polyploidization of HLCs. **A** Representative flow cytometry plots of the width versus height of the 535 channel (PI). Mononucleated diploid [2n], mononucleated tetraploid [4n], binucleated tetraploid (2n + 2n), and binucleated octoploid (4n + 4n) HLCs are shown. Analysis of secreted CYP1A2 and CYP3A4 activity (**B**); secreted cholesterol and triglyceride (**C**); secreted glycogen and urea (**D**); and secreted albumin (ALB) and alpha-fetoprotein (AFP) (**E**). **F** Representative western blotting gels for ALB, AFP, CTNNB1, ERK1/2, and GAPDH. Blue, control HLCs; Green, etoposide-treated HLCs; Orange, RCM-1-treated HLCs. **G** Schematic summary for the effects of FOXM1 and TOP2A inhibition in HLCs. Green arrow, activation; Red bar, inhibition (Created using BioRender.com). In every experiment, *n* = 2 biological replicates (7007 and 7405 cell lines) and 3 independent experiments. In every experiment, etoposide was used at 10 μM and RCM-1 was used at 1 μM for five to twenty-one days. In all experiments, data is mean ± SD. In all experiments, one-way ANOVA with multiple comparisons and Tukey’s correction (p < 0.05).

To evaluate functional HLC maturation, we investigated mononucleated and binucleated polyploidization [37]. In the human liver, 30% of hepatocytes are polyploid because of cytokinesis failure during early postnatal life [29]. Flow cytometry with PI staining revealed that etoposide increased mononucleated polyploidy by 27.6%, whereas RCM-1 led to a 7.4% increase (Fig. 5A). To assess the trade-off between hepatocyte proliferation and metabolic maturation, we assessed HLC metabolic maturation upon inducing cell cycle arrest [38]. After 3 weeks of treatment (D41), we observed enhanced CYP1A2 and CYP3A4 activities with FOXM1 inhibition (~2.5×) (Fig. 5B), as well as CYP1A2 and CYP2B6 trends (Supplementary Fig. S3F). The upregulation of CYP1A2 with RCM-1 may be linked to AHR signaling [39, 40], whereas CYP3A4 upregulation might involve PXR/RXR signaling [41], consistent with our phosphoproteomic findings (Fig. 4I). These pathways are essential for xenobiotic metabolism during terminal differentiation [42]. RCM-1 treatment resulted in increased cholesterol secretion (~1.9×) and decreased triglyceride secretion (~4.2×) in HLCs (Fig. 5C, Supplementary Fig. S3G), indicating enhanced APOB production and effective lipid homeostasis [43, 44]. In assessing hepatocyte endobiotic metabolism, secreted glycogen was higher under RMC-1 treatment, while secreted urea was not significantly different (Fig. 5D). Meanwhile, in assessing hepatocyte damage, secreted ALT levels were higher in the controls, whereas AST levels showed no difference (Supplementary Fig. S3H). ALB levels were not significantly different at D41, while we observed a significant reduction in AFP levels in the treated hepatocytes (Fig. 5E, Supplementary Fig. S3I). Western blotting indicated unchanged levels of ALB, CTNNB1, and ERK1/2, but a notable reduction in AFP in the RCM-1-treated groups (~5.6×) (Fig. 5F).

In summary, we found that long-term TOP2 and FOXM1 inhibition resulted in no cytotoxicity and induced mononucleated hepatocyte polyploidization. In addition, only FOXM1 inhibition was associated with HLC metabolic maturation and fetal protein downregulation, potentially indicating the consistent in vitro terminal differentiation of hiPSC-derived hepatocytes.

## Discussion

We explored the roles of TOP2A and FOXM1 in the terminal differentiation of HLCs – the final developmental stage where fully functional adult hepatocytes are produced -, a critical aspect of liver modeling that current protocols have yet to fully achieve. Inhibition of FOXM1 primed the terminal differentiation of HLCs by inducing cell cycle arrest, polyploidization, and metabolic maturation, while reducing fetal markers in a sustained manner after long-term cell culture maintenance. The transition from the fetal to adult stage is governed by the Wnt/β-Catenin Pathway [45, 46]. FOXM1 inhibition leads to a decrease in Wnt target expression [47] and β-catenin expression, highlighting its significance in hepatocyte maturation. Prolonged FOXM1 inhibition enhanced CYP1A2, CYP2B6 and CYP3A4 activities and improved lipid metabolism. miR-122 is essential for hepatocyte polyploidization and targets FOXM1 [45], indicating that FOXM1 downregulation may contribute to miR-122-dependent terminal hepatocyte maturation. Extended FOXM1 inhibition results in a doubling of mononucleated hepatocyte ploidy, which is linked to the maturation of HLC lipid metabolism. FOXM1 upregulates TOP2A expression by binding to its promoter [48]. TOP2A facilitates cell cycle progression during liver development [49] and its downregulation is associated with hepatocyte maturation [50]. Long-term inhibition of TOP2A increases HLC polyploidization to levels observed in adult livers and enhances ALB expression, whereas short-term inhibition reduces chromatin accessibility and silences cell cycle genes. Overall, the mutual regulation of TOP2A and FOXM1 may be vital for the late-stage maturation of HLCs.

Although we studied FOXM1 and TOP2A in the terminal differentiation of HLCs, postnatal development creates hepatocyte subpopulations via liver zonation [45]. FOXM1 and TOP2A modulation in vitro may produce interzonal-like hepatocytes owing to the lack of metabolic compartmentalization in our system [51]. Thus, pericentral, periportal, and interzonal hepatocyte differentiation require further study. To improve hepatocyte maturation, we proposed targeting FOXM1 and TOP2A using 3D hepatocyte differentiation protocols for a more physiological model, as these enable three-dimensional cell interactions and native extracellular matrix reproduction [52]. Our approach was limited to hepatocytes, excluding interactions between parenchymal and non-parenchymal liver cells. However, iPSCs can produce cholangiocytes, hepatic stellate cells, and Kupffer cells, which are essential for modeling liver disease, and their interactions documented in liver organoid models must be accounted for in the development and disease modeling [53]. Finally, FOXM1 knockout murine models showed abnormal hepatocyte polyploidization, suggesting that FOXM1 modulation requires fine-tuning during late development [54]. Thus, further in vivo studies are needed to validate our findings on FOXM1 and TOP2A modulation of liver development, especially focusing on investigating how structural and numerical chromosomal changes support or not our proposed mechanistic mode through modulating genes at altered loops/compartments that have corresponding transcriptional changes relevant to maturation.

Despite our understanding of cell cycle regulation during hepatocyte terminal maturation, there is a lack of clarity regarding how these mechanisms function during embryonic liver development, regeneration, and cancer. Therefore, we hypothesized that FOXM1 was a key candidate for hepatocyte cell cycle regulation. FOXM1 is crucial for hepatocyte cell cycle entry post hepatectomy, promoting essential proteins such as cyclin A2 and B1 for mitotic progression [55]. FOXM1 promotes hepatocyte proliferation and liver mass restoration through its interaction with Wnt/β-catenin signaling [56]. Although FOXM1 facilitates regeneration, its overexpression can promote liver cancer [57], which is correlated with malignancy and poor prognosis [58]. FOXM1 inhibition using small molecules reduces hepatocellular carcinoma growth [59], and experimental models have shown decreased tumorigenic capabilities [60]. However, given the well-established role of FOXM1 in cell cycle regulation, it remains unclear whether the observed maturation phenotypes that are a direct consequence of FOXM1 suppression or an indirect effect of reduced proliferation, which requires further studies specifically targeting FOXM1 using other inhibitors or methods (miRNA, siRNA, ASOs), as well as specifically inducing cell cycle arrest and evaluating hepatocyte’s maturation phenotype. Thus, investigation of FOXM1 modulation may be critical for understanding liver regeneration and carcinogenesis.

In summary, our results indicated that FOXM1 inhibition in HLCs is associated with enhanced hepatocyte terminal differentiation, characterized by cell cycle arrest, polyploidization, and metabolic maturation.

## Materials and methods

### Treatment with small molecules

For topoisomerase II inhibition, 10 mM etoposide (Sigma-Aldrich, Saint Louis, Missouri, USA) was dissolved in dimethyl sulfoxide (DMSO). For FOXM1 inhibition, 2 mM RCM-1 (Sigma-Aldrich) was dissolved in dimethyl sulfoxide (DMSO) and Etoposide or RCM-1 was diluted in DMSO at 100x a final intended concentration. Next, 100x etoposide or RCM-1 was directly added to the hepatocyte maturation medium and diluted 1:100. Etoposide was predominantly used at a final concentration of 10 μM, whereas RCM-1 was used at a final concentration of 1 μM. The control groups were treated with 1% DMSO in hepatocyte maturation medium. The medium was changed every 48 h, and the treatments were initiated on differentiation day 20 containing either etoposide, RCM-1 or 1% DMSO (control group). Therefore, the drugs or DMSO were replaced during media change every 48 h.

### RNA-extraction, RT-qPCR, and RNA-sequencing analysis

RNA was extracted using a PureLink RNA Mini Kit (Thermo Fisher Scientific). Reverse transcription was performed using the Superscript IV Synthesis Kit (Thermo Fisher Scientific). RT-qPCR was performed using Power SYBR Green PCR Master Mix (Thermo Fisher) on a QuantStudio 5 System. The samples were amplified using primers designed using Primer-BLAST or retrieved from PrimerBank (Supplementary Table 1). Expression analysis was performed using the ∆∆Ct method and normalized to the RPLP0 or GUSB controls. Human Plateable Hepatocytes were used as the PHH controls and were harvested for 24 h. RNA sequencing was performed using the Novogene software. RNA quality was assessed using Bioanalyzer 2100 (Agilent). Library preparation was performed using the TruSeq Stranded mRNA Kit, and sequencing was performed using NovaSeq 6000 (Illumina). The reads were aligned to the human genome hg38 using Hisat2. Differential gene expression analysis was performed using the DESeq2 package in R. The thresholds for differential expression were adjusted P value ≤ 0.05, and |log2(fold change)| ≥ 1. ClusterProfiler was used to test the enrichment of differentially expressed genes. Data visualization was performed using the clusterProfiler and pheatmap packages and the MaGIC Volcano Plot Tool. PPI analysis was performed using the STRING database and term enrichment was performed using the Enrichr web tool.

### ATAC-sequencing analysis

ATAC sequencing was conducted through Novogene. Nuclei were incubated with a Tn5 transposase mix at 37 °C for 30 min. Libraries underwent PCR amplification, were purified using AMPure beads, and evaluated with Qubit. Sequencing was performed on the Illumina Hiseq Platform, producing 150 bp paired-end reads. Reads were trimmed with Skewer, aligned using BWA, and filtered for quality (MAPQ 13) and proper pairing (>18 nt). Peak calling was executed with macs2 using the command ‘macs2 callpeak --nomodel --keepdup all-call summits (v2.1.2). Term enrichment was analyzed with Enrichr, and motifs were identified using HOMER. The ATAC-seq library preparation adhered to the ATAC 2.0 protocol. Cells were fixed with paraformaldehyde, quenched with glycine, and stored at −80 °C. Following lysis, cells were digested with DpnII, labeled with biotin-14-dCTP, and ligated. Libraries were prepared using the KAPA HyperPlus Kit with biotin enrichment. Data processing was carried out with Juicer and Cooltools. Normalized maps were analyzed using FANC, and compartmentalization was assessed with cooltools saddle functions. A/B compartments were assigned through eigenvector decomposition.

### Proteomic analysis

For proteomic analysis, samples were lysed using buffer containing 50 mM HEPES (pH 8), 1% SDS, 1% sodium deoxycholate, 5 mM EDTA, and 50 mM NaCl, with Halt Protease and Phosphatase Inhibitor Cocktail. Proteins were quantified using the Pierce BCA Protein Assay Kit, and 250 μg in 100 μL was digested using a modified semi-automated SP3 protocol. Phosphopeptides were enriched using titanium dioxide beads (Titansphere, Gl Sciences, Japan), according to the manufacturer’s instructions. LC-MS/MS analyses were performed using a nano LC UltiMate 3000 system connected to an Exploris 240 hybrid quadrupole-orbitrap tandem mass spectrometer (Thermo Fisher Scientific, Bremen, Germany) equipped with an EASY-spray source operating in positive ion mode. Spectral data were searched against an in silico human spectral library (Uniprot Swissprot database downloaded on August 06, 2024, which contained 20,447 entries) using DIA-NN 1.9.1. FragPipe-Analyst was used for visualization, Enrichr for term enrichment, and X2K web tool for regulatory networks.

### Flow cytometry analysis

For flow cytometry analysis, the cells were dissociated using TrypLE Express Enzyme (Thermo Fisher Scientific). The cells were fixed and permeabilized using the Fix & Perm Cell Fixation and Permeabilization Kit (Thermo Fisher Scientific), according to the manufacturer’s instructions. The samples were blocked with 5% BSA in PBS [1x] for 1 h and then incubated with primary antibodies (listed in Supplementary Table 3) at 4 °C for 1 h. After washing with PBS [1x], the samples were incubated with Alexa Fluor-conjugated secondary antibodies (Supplementary Table 3) at 4 °C for 1 h. The samples were analyzed using a FACS Aria III flow cytometer (BD Biosciences) with 20,000 events per group. For ploidy analysis, samples were dissociated with TrypLE Express (Thermo Fisher), fixed with 70% ethanol at –20 °C for 2 h, and DNA was stained using FxCycle PI/RNase Staining Solution (Thermo Fisher) with 100 U/μL RNase I for 30 min at 37 °C, acquiring 500,000 cells per group. For DNA fragmentation and damage assays, we used the Click-iT TUNEL Alexa Fluor 488 kit and an anti-phospho-H2AX antibody (Supplementary Table 3). The CellEvent Senescence Green Flow Cytometry Assay Kit (Thermo Fisher Scientific) was used for senescence evaluation. Data were analyzed using the FlowJo V10 software.

### Toxicity assays

For the cytotoxicity assays, HLCs were differentiated into 96-well plates. To assess cell viability, intracellular ATP levels were measured using the CellTiter-Glo Luminescent Cell Viability Assay Kit (Promega, G7571), according to the manufacturer’s instructions. To assess toxicity, 200 μL of the supernatant was collected and lactate dehydrogenase (LDH) was measured using the CyQUANT LDH Cytotoxicity Assay (Thermo Fisher, C20301), according to the manufacturer’s instructions. Both assays were normalized using a blank negative control (hepatocyte maturation medium) and death-positive control (5% Triton-X100 treatment for 15 min).

### Quantification and statistical analysis

Statistical analyses were performed using GraphPad Prism 8 with an unpaired two-tailed Student’s t test, one-way analysis of variance (ANOVA), and post hoc Tukey’s test. Statistical significance was set at P < 0.05. n represents number of biological replicates and independent experiments. G*Power software was used to determine the minimum sample size for data collection to achieve α = 0.05 and power =p0.8.

## Supplementary information

Supplemental material

## Data Availability

The RNA-seq, ATAC–seq, and Hi-C data reported in this study have been deposited in the NCBI Gene Expression Omnibus (GEO) with accession numbers GSE298619, GSE298820, and GSE298819, respectively. The proteomic data reported in this study have been deposited in the ProteomeXchange Consortium via the PRIDE partner repository under accession number PXD064121.
