# Supplementary material for: FOXM1 inhibition primes terminal differentiation of human iPSC-derived hepatocytes
Source: Cell Death Discov. 2026 Jul 8;12:394. doi: 10.1038/s41420-026-03178-9 (PMC13631359; doi:10.1038/s41420-026-03178-9)
Supplement: Supplementary file 1 [file 41420_2026_3178_MOESM1_ESM.pdf]

Supplementary Materials for

FOXM1 inhibition primes terminal differentiation of human iPSC-derived hepatocytes

Kayque Alves Telles-Silva *et al.*

*Corresponding author: mayazatz@usp.br.

This PDF file includes:

Supplementary Text
Figs. S1 to S3
Tables S1 to S4
References (1 to 4)

Supplementary Text

hiPSC cell maintenance

All cell culture experiments used four human induced pluripotent stem cells (hiPSCs) from healthy male participants. Informed consent was obtained from all subjects. WTC-11, a characterized control cell line (1), was purchased from the NIGMS Repository. Three other lines (7007, 7405, and 8799) were established at the Human and Stem Cell Research Center. Every cell line was tested for mycoplasma contamination every three months. The sample size was primarily determined by feasibility (available population and resources). We then conducted a post hoc power analysis indicating that a total sample of N = 3 provides approximately 80% power ($\alpha = 0.05$, two-sided) to detect a pre-specified effect size, which we considered biologically meaningful based on prior literature. We evaluated the variance between the groups by inspecting residual plots and formally tested using Levene's test ($p > 0.05$), indicating no evidence of heteroscedasticity. hiPSCs were maintained on 10 μg/cm2 hESC-qualified Matrigel (Corning)-coated plates using Essential 8 Medium (Thermo Fisher) with 100 μg/mL Normocin (InvivoGen). The medium was replaced daily. Cells were split every 3-4 days using Accutase (Gibco), seeded at 3×10^4 cells/cm2 in Essential 8 medium with 5 μM Y-27632 (Sigma-Aldrich) for 24h, and maintained until passage 30 (p30).

Hepatocyte differentiation

Hepatocyte differentiation was performed as described previously (60). Single-cell hiPSCs were seeded in Matrigel-coated 6-well plates at 7 × 10^5 cells/well in Essential 8 medium with Y-27632. At 40% confluency on day 2, the medium was changed to RPMI 1640 with 2% B-27 minus Vitamin A 100 ng/mL Activin A 25 ng/mL Wnt3a, and 100 μg/mL normocin. The medium was changed daily for three days and then extended without Activin A two more days. On day 6, the medium was switched to Knockout DMEM with 20% Knockout Serum Replacement, 1% DMSO, 0.5% Glutamax, 1% NEAA, 0.1 mM beta-mercaptoethanol, and 100 μg/mL normocin, and changed daily from days 6 to 8 and on day 10. On day 11, the medium was switched to the Hepatocyte Culture Medium BulletKit with 20 ng/mL oncostatin M and 100 μg/mL normocin, changed every 48h until day 20.

Transmission electron microscopy

For transmission electron microscopy (TEM), the cells were dissociated using TrypLE Express (Thermo Fisher Scientific) and pelleted. The Pellets were fixed in 2.5% (v/v) glutaraldehyde in PBS (1x) for 2 h at 4°C, post-fixed in 1% OsO_4 for 1 h at 4°C, and stained overnight in 1% aqueous uranyl acetate. The pellets were sequentially dehydrated in 30%, 70%, and 100% ethanol. The samples were then embedded in epoxy resin. Ultrathin sections (70 nm) were obtained using an ultramicrotome, collected on nickel grids, and double stained with uranyl acetate and lead citrate. Micrographs were obtained using a JEOL JEM 1010 electron microscope (80 kV).

Comet assay

A comet assay was performed to evaluate genomic DNA damage. The cells were resuspended in agarose, distributed on slides previously coated with the same material, and allowed to solidify for 30 minutes at 4°C. After hardening, the slides were treated with a lysis

buffer at 4°C for at least 40 min. After cell membrane lysis and DNA exposure, slides were immersed in an alkaline unwinding solution for 20 min at room temperature. Electrophoresis was conducted in a denaturation buffer at 21 Volts and 0.3 A for 30 min. Staining was performed with SYBR Green (Thermo Fisher Scientific). Images were captured using an Axiovert 200 fluorescence microscope, and 100 nuclei per condition were analyzed using LUCIA Comet Assay software (Laboratory Image). The head/tail DNA ratio (tail momentum) was calculated by using 100 comets on each slide for each sample.

Hi-C analysis

To prepare the Hi-C library, 1–5 × 10^6 cells were dissociated using trypsin for 15 min, centrifuged at 500 × g for 5 min, washed with PBS, and fixed with 1% paraformaldehyde for 10–12 min. After glycine quenching, cells were pelleted, washed, and stored at − 80°C. Thawed cells were lysed and digested with 300 U of DpnII. The DNA ends were labeled with biotin-14-dCTP using Klenow fragments and ligated with T4 DNA ligase. Crosslinks were reversed using Proteinase K at 65°C, followed by ethanol precipitation. Unligated biotinylated ends were removed using T4 DNA polymerase. NGS libraries were prepared using the KAPA HyperPlus Kit, with biotin-labeled products enriched using Dynabeads MyOne Streptavidin C1, before amplification. Sequencing used 150 bp paired-end reads. Hi-C data were processed using Juicer and Cooltools .hic and .cool files. Quality metrics were computed using a modified Juicer script. Differentially expressed gene loci were analyzed using normalized Hi-C maps and were visualized using the FANC library. Compartmentalization strength was assessed using cooltools saddle and saddle_strength, with eigenvector decomposition for A/B compartment assignment.

To compare TAD boundary strength between conditions (PHH, control, and Etoposide-treated), we generated scatter plots for each pairwise comparison. Insulation scores were calculated at 25 kb resolution using the cooltools insulation function. The resulting .bed files containing insulation scores were intersected with .bedpe files of TAD boundaries to assign a score to each boundary. For each pairwise comparison, boundaries showing an insulation score difference greater than 3 standard deviations from the main distribution were considered outliers. These outlier regions were subsequently visually validated using Juicebox.

We used two independent sets of chromatin loops in this study. The first set was identified using HiCCUPS (2). Loop length was calculated as the genomic distance between the centroids of loop anchors. For the Venn diagram, loop anchor regions were extended by 10 kb on each side, and pairwise intersections were performed between loops identified in Control, PHH, and Etoposide-treated samples. Loop strength was evaluated using a custom script that calculated the average value of pixels in the balanced submatrix around each loop, derived from matrices normalized with cooltools balance. Additionally, z-scores were computed to evaluate the median loop strength in PHH samples deviated from the distribution observed iPSC-derived. The second set of loops was identified using the cooltools dots (ref 2), which detect focal enrichments in contact matrices after estimating expected contact frequencies with cooltools expected-cis. Aggregation of contact signals over loop regions was performed with coolpup.py, followed by visualization using plotpup.py (3).

The contact probability as a function of genomic distance, P(s), was analyzed to assess chromatin scaling lows. Smoothed and aggregated contact frequency curves were computed using cooltools expected-cis with Gaussian smoothing (σ = 0.1). Log-log plots of P(s) were

generated to visualize contact decay profiles, and first derivatives were computed to highlight changes in the scaling slope across genomic distances (4).

Immunostaining

For immunostaining, cells were fixed with 4% paraformaldehyde for 15 min and washed three times with PBS (1x). The samples were permeabilized with 0.01% Triton X-100 in PBS (1x) for 30 min, followed by blocking with 5% bovine serum albumin (BSA) in PBS (1x) for 1 h. The samples were incubated with primary antibodies (listed in Supplementary Table 2) at 4 °C overnight with gentle shaking. The samples were then incubated with secondary antibodies (Supplementary Table 2) at 4 °C for 1 h at room temperature. Nuclei were counterstained 4°Cth DAPI (Sigma) for 1 min at room temperature. Samples were imaged using a Zeiss LSM 800 confocal microscope (Zeiss). Images were analyzed using ImageJ software (v2.0.0).

Western blotting

For western blotting, cells were lysed using RIPA Buffer with Protease and Phosphatase Inhibitor Cocktail (Thermo Fisher). Proteins were quantified using a Pierce BCA Protein Assay Kit. Twenty micrograms of protein was denatured using NuPAGE LDS Sample Buffer at 70°C for 10 min. Precision Plus Protein Western C standards were used as size standards. Proteins were loaded onto Novex Tris-Glycine Mini Protein Gels at 120 V for 1 h and then dry-transferred to iBlot 2 PVDF membranes using an iBlot2 Gel Transfer Device. The membranes were blocked with 5% bovine serum albumin (BSA) in TBST for 1 h at room temperature. The membranes were incubated with primary antibodies (Supplementary Table 4) at 4°C overnight using GAPDH as the endogenous control. After washing with TBST, the membranes were incubated with horseradish peroxidase-conjugated secondary antibodies for 1 h. Chemiluminescence was detected using the Amersham ECL Prime Reagent. Protein bands were imaged using the ChemiDoc MP System and analyzed using the ImageLab software.

Metabolite assays

Total cholesterol and triglyceride secretion was measured by collecting 1 mL of the supernatant from HLCs cultured in HCM and stored at −80 °C until use. The supernatant was assayed using the HDL and LDL/VLDL Cholesterol Assay Kit (Abcam, ab65390) and Triglyceride Assay Kit (Abcam, ab65336) following the manufacturer's instructions.

Protein secretion and metabolic activity assays

Human ALB and AFP secretion were measured by collecting 1 mL of the supernatant from HLCs cultured in HCM and stored at −80 °C until use. The supernatant was assayed using the Human Albumin ELISA Kit (Thermo Fisher, EHALB) and Human Alfa Fetoprotein ELISA Kit (Abcam, ab108838) according to the manufacturer's instructions. Similarly, metabolic activity was measured using the Alanine Transaminase (ALT) Activity Assay (Abcam, ab105134), Aspartate Aminotransferase (AST) Activity Assay (Abcam, ab105135), P450-Glo CYP1A2 Induction/Inhibition Assay (Promega, V8421), and P450-Glo CYP2B6 Assay (Promega, V8321), according to the manufacturer's instructions.

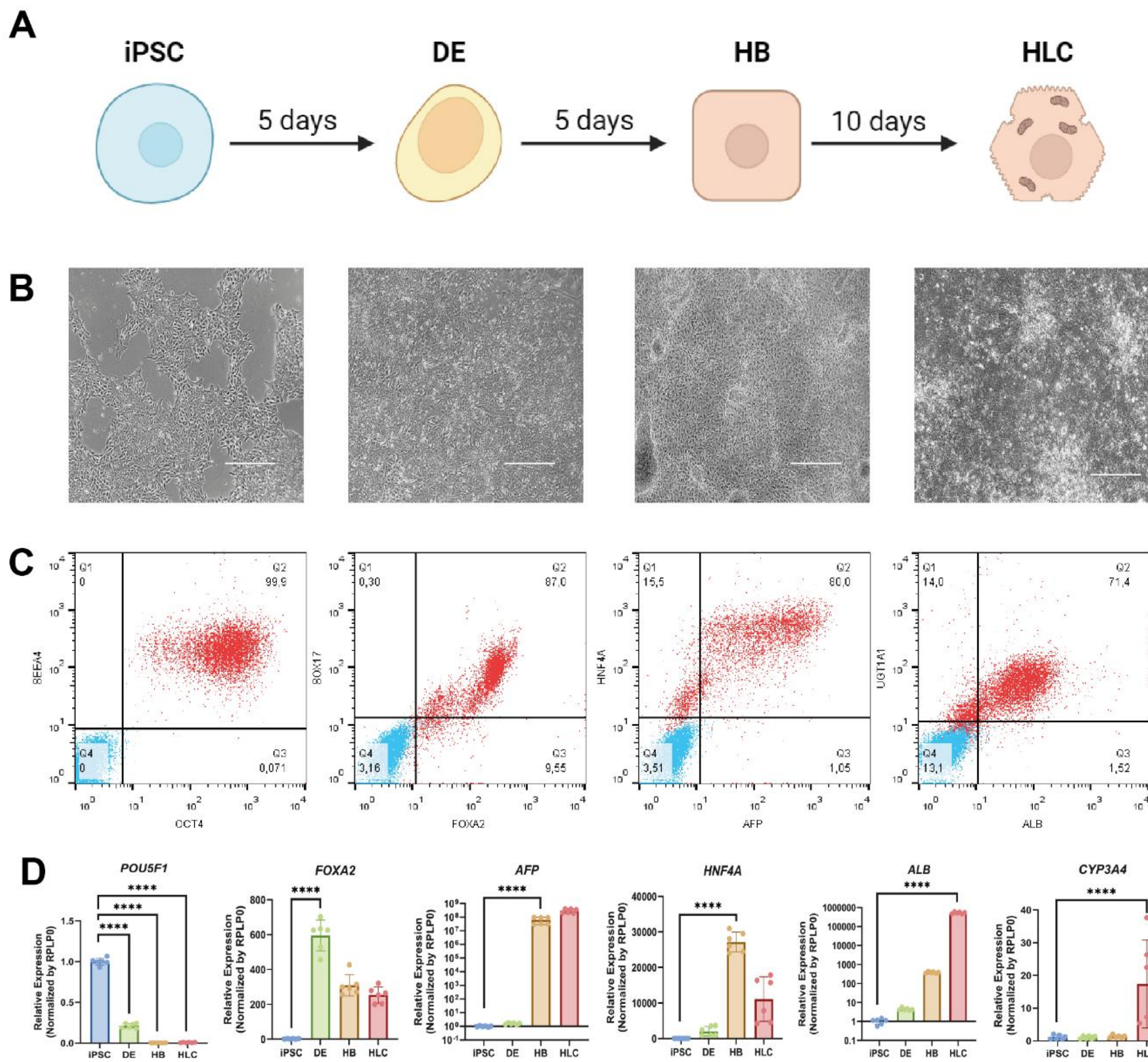

Fig. S1: Differentiating iPSCs into HLCs. A) Schematic for iPSC differentiation into HLCs. iPSC, induced pluripotent stem cell; DE, definitive endoderm; HB, hepatoblast; HLC, hepatocyte-like cell. **B**) Brightfield images of differentiation progression from iPSCs to HLCs (scale bar = 400 µm). **C**) Stepwise flow cytometry characterization of iPSC differentiation into HLCs for hiPSC markers OCT3/4 and SSEA4; pan-endoderm markers FOXA2 and SOX17; hepatoblast markers AFP and HNF4A; and pan-hepatocyte markers ALB and UGT1A1. Blue, negative isotype-stained control; Red, cell from the respective differentiation stage ($n = 2$, biological replicates and 3 independent experiments). **D**) Stepwise RT-qPCR characterization of iPSC differentiation into HLCs for the iPSC marker *POU5F1*; the endoderm marker *FOXA2*; the hepatoblast markers *AFP* and *HNF4A*; and the hepatocyte markers *ALB* and *CYP3A4* (Data is mean ± SD; ****$p<0.001$; $n = 2$, biological replicates (7007 and 7405 cell lines) and 3 independent experiments). In D, one-way ANOVA with multiple comparisons and Tukey's correction.

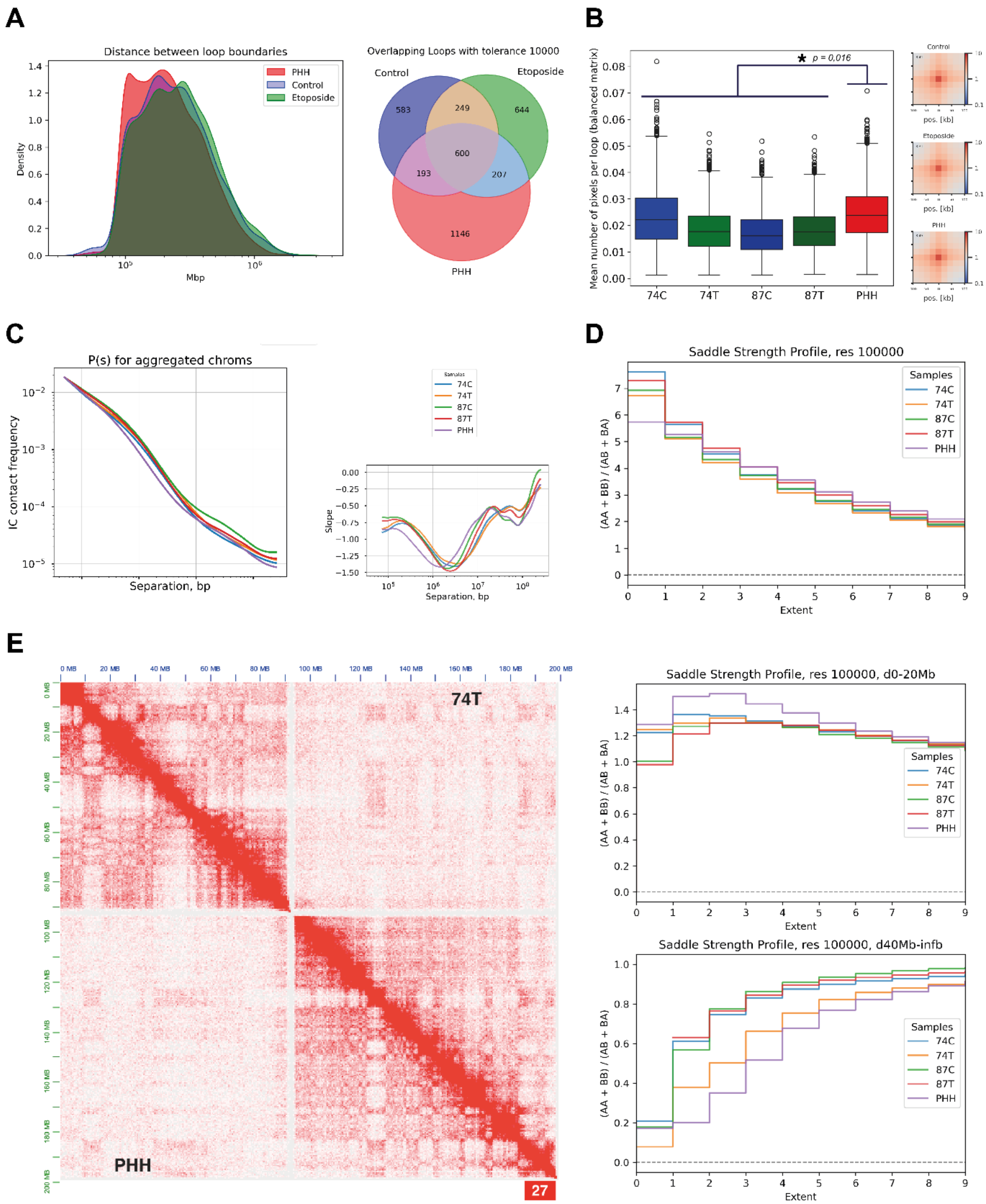

Fig. S2: Hi-C analysis of etoposide-treated HLCs. A) Loop characteristics and overlap across PHH, control, and etoposide-treated samples. Left, distribution of loop lengths (measured as the genomic distance between loop anchors) for each condition. Right, Venn diagram of overlapping chromatin loops across conditions, using a 10 kb tolerance for anchor matching. **B**) Aggregate loop signal and strength. Left, boxplot of loop strength values computed from balanced contact matrices ($z = 2.14$, $p = 0.016$). Right, average pileup plots of chromatin loops. **C**) Contact probability decay curves and their derivatives for aggregated chromosomes. Left, P(s) plots

showing the probability of chromatin contacts as a function of genomic separation. Right, first derivative of the log-transformed P(s) curves. **D**) Compartmentalization strength profiles in 100 Kb resolution. **E**) Compartmentalization between PHH and HLCs at different genomic distances. Left, contact maps of chromosome 3 from PHH vs 74T. Right, saddle strength profiles for mid-range interactions (0–20 Mb) and long-range interactions (>40 Mb) at 100 kb resolution. 74C and 87C, vehicle controls; 74C and 87T, etoposide-treated HLCs; PHH, primary human hepatocytes ($n = 2$, biological replicates (7405 and 8799 cell lines); data displayed as mean ± SD). Etoposide was used at 10 μM for 5 days, with reposition every other day.

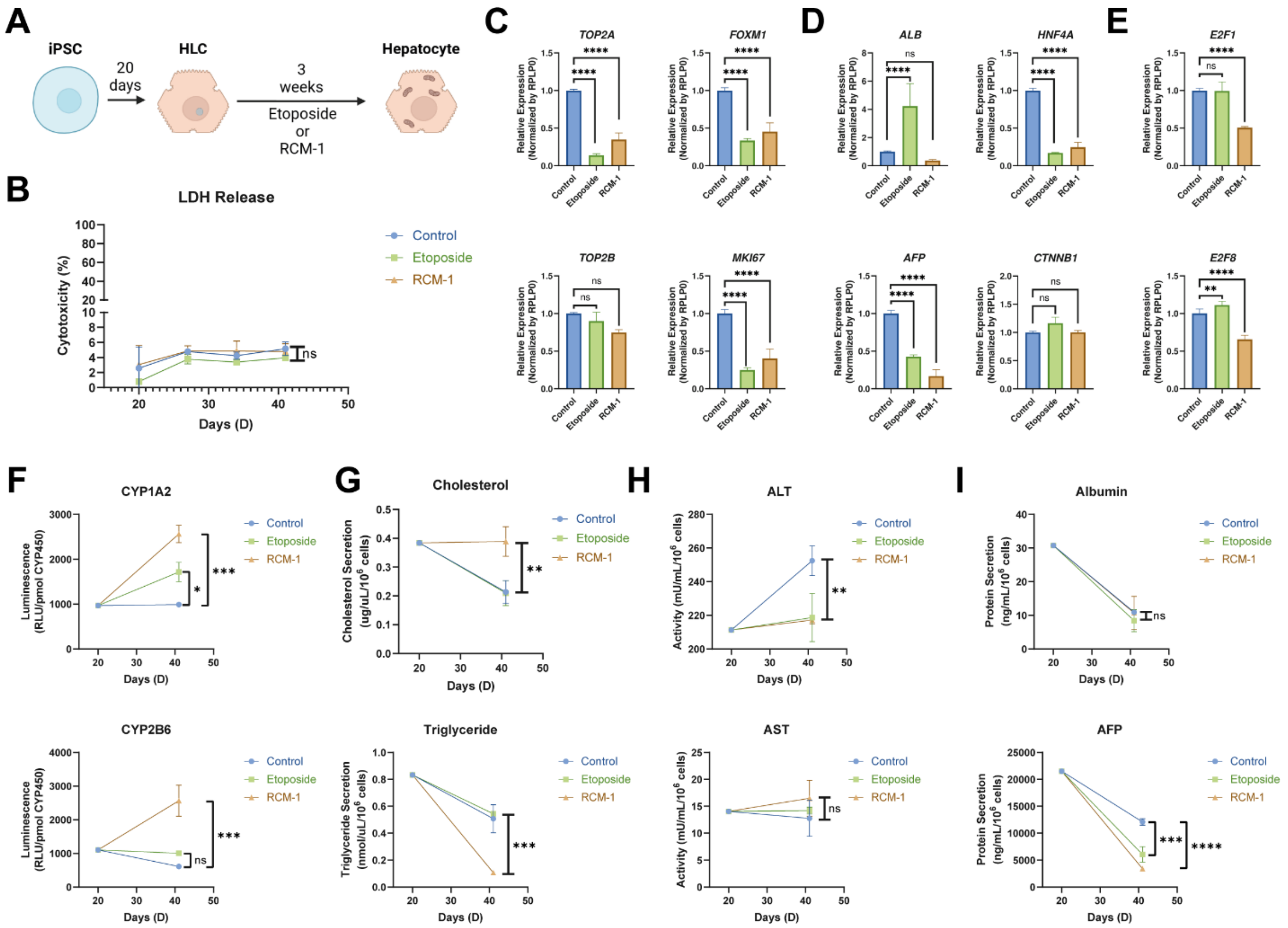

Fig. S3: Characterization of long-term exposure to RCM-1 and etoposide. A) Schematic of long-term treatment of HLCs with etoposide or RCM-1. **B**) Cytotoxicity assay (LDH release) of HLCs with RCM-1, etoposide, or 1% DMSO (control) for 41 days. **C-E**) RT-qPCR of *TOP2A*, *TOP2B*, *FOXM1*, and *MKI67* (**C**); *ALB*, *AFP*, *HNF4A*, and *CTNNB1* (**D**); or *E2F1* and *E2F8* (**E**) of control, etoposide- and RCM-1-treated HLCs. **F-I)** Analysis of secreted CYP1A2 and CYP2B6 activity (**F**); secreted cholesterol and triglyceride (**G**); secreted alanine aminotransferase (ALT) and aspartate aminotransferase (AST) activity (**H**); and secreted albumin (ALB) and alpha-fetoprotein (AFP) (**I**). In every experiment, $n = 2$ biological replicates (7007 and 7405 cell lines) and 3 independent experiments. In all experiments, data is mean ± SD. In all experiments, one-way ANOVA with multiple comparisons and Tukey's correction ($p<0.05$). Etoposide was used at 10 µM for 21 days, with reposition every other day. RCM-1 was used at 1 µM for 21 days, with reposition every other day.

Table S1. Primer Sequence for RT-qPCR

Gene	Forward	Reverse
AFP	CAGGGTGTTTAGAAAACCAGCTAC	TGCAGCAGTCTGAATGTCCG
ALB	CTCGGCTTATTCCAGGGGTG	AAAGGCAATCAACACCAAGGC
CDKN1A	TGTCTTGTACCCTTGTGCCTC	CGTTTGGAGTGGTAGAAATCTGTC
CENPF	AGATGGAGTCCAAGTTGGCG	CGGCCTTGAATAGCATCTTCTG
CTNNB1	CCTGTTCCCCTGAGGGTATTTG	ACTCCATCAAATCAGCTTGAGTAGC
CYP3A4	ACCTTGTAAAGAAACACAGATCCC	TCAGGCTCCACTTACGGTG
E2F1	CAGGAGGTCACTTCTGAGGAGG	GACAACAGCGGTTCTTGCTCC
E2F8	GGAATTTCGGGCAGCTTCTG	TGAGGCGTTGACACCAAAAAC
FOXA2	TGCACTCGGCTTCCAGTATG	CATGTTGCTCACGGAGGAGT
FOXM1	AAACGGGAGACCTGTGATGG	ATCTCTTGCTTGATGCTGCG
GLB1	TGTGCAGAGTGGGAAATGGG	CTGCCAGGTAATCTGGGTCG
GUSB	TTCCTATGCCATCGTGTGGG	TGGCGATAGTGATTCGGAGC
HNF4A	TGGACAAAGACAAGAGGAACC	ATAGCTTGACCTTCGAGTGC
MKI67	GGATCGTCCCAGTGGAAGAG	GTCTCGTGGGCCACATTTTC
PLK1	AGTGTCAATGCCTCCAAGCC	TCACAGAGCTGATACCCAAGG
POU5F1	AATTTGTTCCTGCAGTGCCC	CACACTCGGACCACATCCTTC
RPLP0	CATATCCGGGGGAATGTGGG	CAGCAGCTGGCACCTTATTG
TOP2A	ACCAAGAATCGCCGCAAAAG	TCTCCCCCTTGGATTTCTTGC
TOP2B	TCTCAGAAGTCAGAAGATGATTCAG	TCTGTTTCAGACCAAATGATGGTG

Table S2. Antibodies for Immunostaining

Antibody	Catalog Number	Host Species	Assay Dilution
Anti-FOXM1	711695	Rabbit	1:100
Anti-phospho-FOXM1 (Thr600)	PA5-105625	Rabbit	1:100
Goat Anti-Rabbit Alexa 546	A11010	Goat	1:1,000

Table S3. Antibodies for Flow Cytometry

Antibody	Catalog Number	Host Species	Assay Dilution
AFP	PA5-21-004	Rabbit	1:100
ALB	ab106582	Chicken	1:100
FOXA2	ab60721	Mouse	1:100
HNF4A	ab41898	Mouse	1:100
OCT3/4	ab19857	Rabbit	1:100
SOX17	703063	Rabbit	1:100
SSEA4	ab16287	Mouse	1:100
UGT1A1	ab129729	Mouse	1:100
γH2AX	MA5-33062	Rabbit	1:100
Donkey Anti-Mouse Alexa 546	A10036	Donkey	1:1,000
Goat Anti-Mouse Alexa 488	11001	Goat	1:1,000
Goat Anti-Rabbit Alexa 488	11034	Goat	1:1,000
Goat Anti-Rabbit Alexa 546	A11010	Goat	1:1,000
Goat Anti-Chicken Alexa 488	A0001	Goat	1:1,000

Table S4. Antibodies for Western Blotting

Antibody	Catalog Number	Host Species	Assay Dilution
AFP	ab133617	Rabbit	1:1,000
ALB	ab207327	Rabbit	1:1,000
CTNNB1	9582P	Rabbit	1:1,000
ERK1/2	4695S	Rabbit	1:1,000
GAPDH	PA1-987	Rabbit	1:1,000
SMAD1	6944P	Rabbit	1:1,000
YAP/TAZ	8418	Rabbit	1:1,000
TOP2A	ab219320	Mouse	1:1,000
Goat Anti-Rabbit HRP	7074S	Goat	1:5,000
Goat Anti-Mouse HRP	7076S	Goat	1:5,000
